# Experiments on hydraulic jumps over uneven bed for turbulent flow modelling validation in river flow and hydraulic structures

**DOI:** 10.1038/s41597-024-03135-0

**Published:** 2024-03-26

**Authors:** Francisco Nicolás Cantero-Chinchilla, Oscar Castro-Orgaz, Sk Zeeshan Ali, Subhasish Dey

**Affiliations:** 1 University of Cordoba, Hydraulic Engineering Area, Rabanales Campus, Leonardo da Vinci Building, 14071 Córdoba, Spain; 2 Indian Institute of Technology Hyderabad, Department of Civil Engineering, Telangana, 502285 India; 3 Indian Institute of Technology Jodhpur, Department of Civil and Infrastructure Engineering, Jodhpur, Rajasthan 342030 India; 4 Department of Hydraulic Engineering, State Key Laboratory of Hydro-Science and Engineering, Tsinghua University, Beijing, 100084 China

**Keywords:** Civil engineering, Hydrology, Water resources

## Abstract

This study presents a comprehensive dataset comprising multiple data packages derived from laboratory experiments on steady and unsteady hydraulic jumps interacting with a large-scale Gaussian-shaped bed obstacle in an open-channel flume. The primary objective was to accurately measure the impact of hydraulic jump on the free surface and the bed pressure along the obstacle, ensuring the transferability of the results. A multi-process method was followed: designed experiments were recorded, images were postprocessed, and water level data were digitalized. For steady conditions, the bed pressure along the obstacle were measured by piezometers. The repository data are organized and provided in a single package, supplemented by a second package containing panoramas for each experimental time instant and graphical representations of the data, facilitating rapid evaluation of the outcomes. This study provides versatile data that can be utilized in various ways, particularly for fluvial model validation and studying turbulence-driven phenomena in open-channel flows. The detailed methodology presented herein can contribute to the advancement of enhanced laboratory techniques to study similar flow problems.

## Background & Summary

Environmental shallow water flows in fluvial streams exhibit complex conditions arising from the interaction of flow, bathymetry, and instream obstacles. These unique conditions are often characterized by the development of large vertical flow accelerations and sudden changes in the flow regime, leading to turbulence-driven hydraulic jumps as the flow transitions from supercritical to subcritical conditions. Consequently, the fluid pressure departs from the hydrostatic law due to the curvilinear streamlines in the flow field^1,2^. In the context of integrated watershed management, accurate modelling of the turbulent flow phenomena in the shallow water framework is crucial for reliable flood level estimations and resilient flow structure designs^3,4^. Therefore, the availability of experimental databases that characterize these unique flow phenomena is essential for the development and validation of robust shallow water models, as frequently conducted in the past with ad-hoc experiments and the production/testing of three-dimensional (3D) RANS or LES models^5–9^. In fact, the generation of such experimental databases is an essential prerequisite for the advancement of efficient management techniques across various hydro-environmental disciplines^10–12^.

In shallow water modelling, depth-averaged models have gained popularity because of their computational efficiency and ease of implementation, particularly when dealing with river-scale applications, as compared to the three-dimensional models^3,13,14^. These models, based on the hydrostatic pressure distribution assumption, are called *shallow water equations* (SWE) models. Numerical methods are routinely employed to solve steady and unsteady flows governed by the SWE equations. However, accurately capturing turbulence-induced flow processes, such as hydraulic jumps, presents a significant challenge within the SWE framework. To address this, turbulence closure models, including zero equation, one equation, and two equation *k*-*ε* models, are commonly used^15^, where *k* is the turbulent kinetic energy (TKE) and *ε* is the TKE dissipation rate. The turbulence closure models rely on various parameters that govern the TKE generation and dissipation, requiring appropriate tunning^3,16^. In order to investigate accurate estimators for these parameters and to benchmark their performances, it is crucial to utilize experimental data that provide precise and detailed measurements of free surface and pressure levels in both steady and unsteady conditions, e.g., data of moving and steady hydraulic jumps.

The occurrence of hydraulic jumps in fluvial streams or canals is often accompanied by the interaction of the flow with singular bed configurations, such as step-pool erosive formations or instream obstacles, like dunes or weirs. The significant vertical deceleration of flow within hydraulic jumps emphasizes the importance of studying their interaction with flows over obstacles, by producing experimental databases that accurately describe hydraulic jumps under real conditions. Consequently, the non-hydrostatic pressure distribution prevails in the vertical direction. In this context, improved SWE models that account for the non-hydrostatic behaviour of the flow become relevant, particularly when the vertical length scale is comparable with the horizontal length scale^2^. However, the development of such models offers mathematical challenges, as it involves complex vertically integrated governing equations. Validating these sophisticated models requires accurate fluid pressure data. Nonetheless, due to the inherent difficulties in reproducing these turbulent phenomena, hydraulic jumps have extensively been studied in the literature under controlled experimental conditions, often simulating jumps occurring downstream of gate opening on horizontal or inclined plane beds^17–19^. Hydraulic jumps over uneven beds are rarely investigated experimentally in spite of the occurrence in real life flow conditions.

The objective of this study is to perform detailed laboratory experiments on hydraulic jumps caused by the interaction of open-channel flows with an obstacle over uneven beds, under both steady and unsteady conditions. Accurate measurements of the free surface level for both steady and unsteady hydraulic jumps and the bed pressure for steady jumps were obtained under controlled laboratory settings. The experiments were carried out in a 15 m long and 0.405 m wide open-channel flume with an uneven bed and an instream Gaussian-shaped bed obstacle. Careful measures were taken during the experiments to minimize undesirable flow disturbances. A monitoring system was used to capture images, which were postprocessed for data extraction. Piezometers were used to measure the bed pressure along the Gaussian-shaped obstacle. Part of this dataset was utilized by Castro-Orgaz *et al*.^20–22^ and Gamero *et al*.^23^ for the validation of a Boussinesq-type SWE model and a variational VAM model, respectively. The postprocessed data used in their study were reported as supplementary material, aiming at to facilitate their utilization by researchers.

This study presents a total of 8 experiments conducted under steady flows and 8 experiments under unsteady flows, all involving hydraulic jumps. Accurate characterization of the free surface level was performed at multiple time instants for each experiment. Additionally, detailed documentation of the bed pressure readings along the Gaussian-shaped obstacle was made for the steady flow experiments. The experimental setup involved variations in upstream heads and discharges, a rapid opening of sluice gate to produce dam-break like flows, and operation of a tailgate. This study provides comprehensive explanations of the methods employed during the experimental campaign and the subsequent postprocessing procedures, ensuring the reproducibility of the experiments. Furthermore, the captured images from the experiments are made available for reuse by the scientific community. The uniqueness of this dataset lies in its combination of open-channel flows over an instream obstacle and an uneven bed, involving moving hydraulic jumps with dispersion, reflection and obstacle interaction, mimicking similar flow phenomena in canals and rivers. The utilization of this dataset available in^24^ enables modellers and researchers to validate new computational models and to enhance the understanding of turbulent phenomena in shallow water environments.

## Methods

### Physical model description

**Fig. 1:**
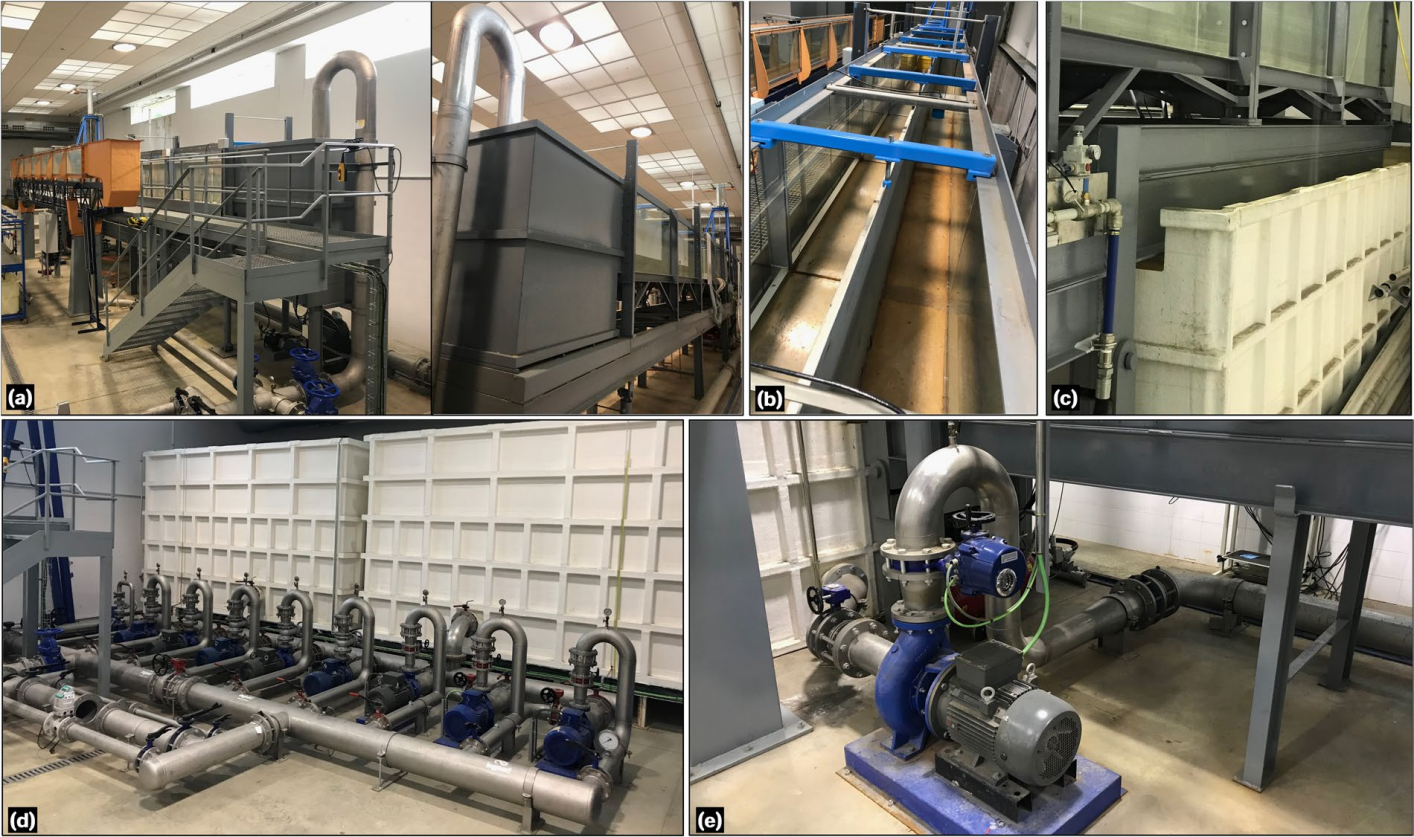
Photographs of the flume facility: (**a**) general view, (**b**) width division wall, (**c**) cantilever and tailwater reservoir, (**d**) headwater reservoirs and pumps, and (**e**) recirculating pump and pipe system.

**Fig. 2:**
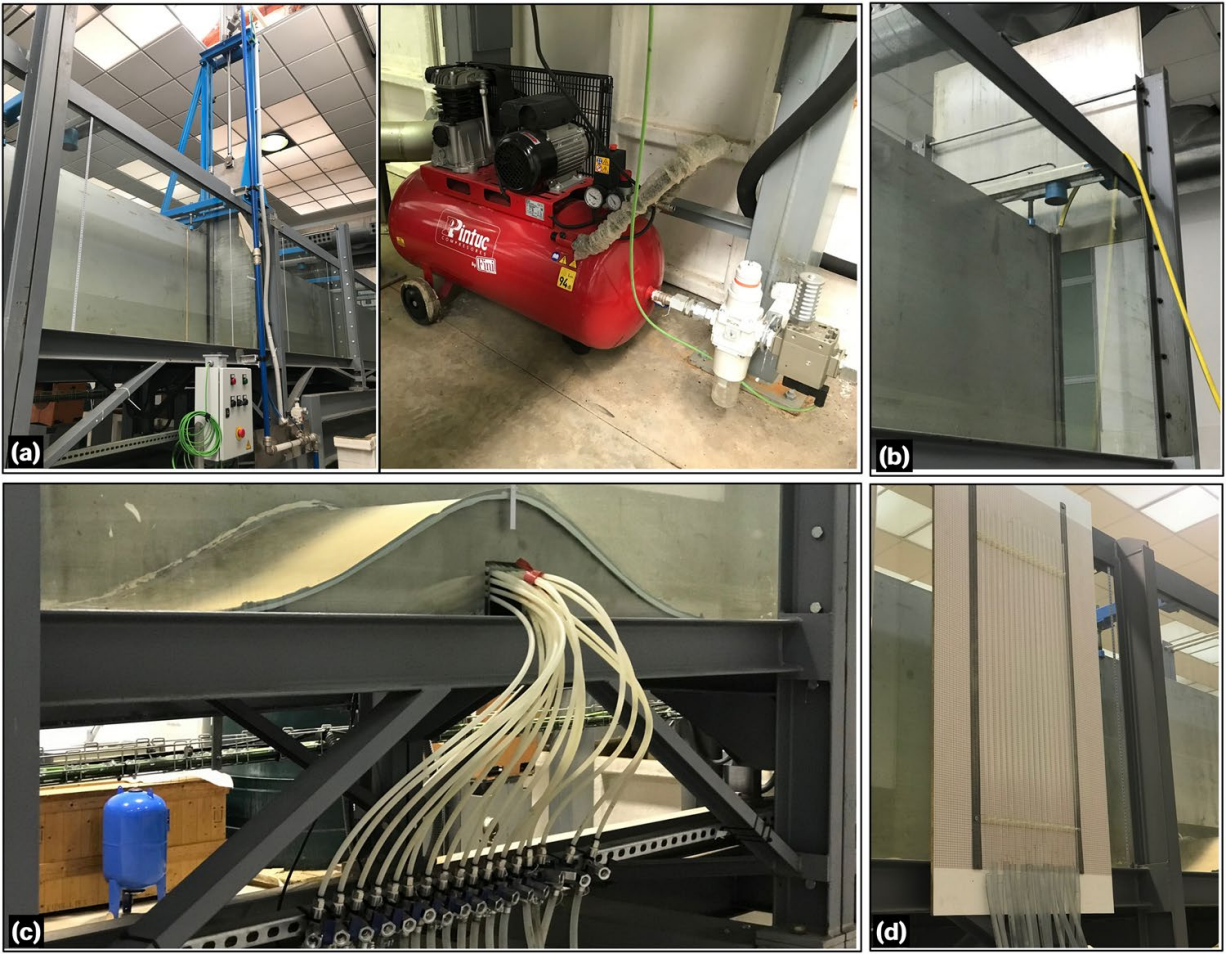
Photographs of the flume devices: (**a**) rapid-release sluice gate, (**b**) tailgate, (**c**) instream Gaussian-shaped bed obstacle, and (**d**) piezometric tubes.

The experiments were conducted in a flume facility (Figs. 1a, 2) located at the laboratory of hydraulics of the University of Cordoba (UCO), Spain. The flume is a tilting flume with a measuring cross-section 1 m wide and 1 m high. However, for the experiments, the width was reduced to 0.405 m using a movable division wall along the 15 m length of the flume (Fig. 1b). To allow visual inspection, a lateral flume wall was installed, which included 8 windows having 1.875 m length and 1 m height. The coordinates for the corners of each window, starting from the flume inlet, are provided in Table 1. Structurally, the flume was supported by fixed metal pillars from the tilting point [located at *x*_*jo*_ = 9.634 m (Fig. 2)] upwards, while the section from the tilting joint to the end of the flume functioned as a cantilever. The portion of the flume extended over the tailwater reservoir, enabling the recirculation of flow (Fig. 1c). After the flume bed parametrization, the expression describing the cantilever section is:

1 $${z}_{cant}=-1{0}^{-5}{\left(x-{x}_{jo}\right)}^{2}\left[{\left(x-{x}_{jo}\right)}^{2}-4\left(x-{x}_{jo}\right)\left(L-{x}_{jo}\right)+6{\left(L-{x}_{jo}\right)}^{2}\right]$$

where *z*_*cant*_ is the local bed level of the cantilever section below the plane flume bed (i.e., with the residual slope), *L* is the total length of the flume, and *x* is the longitudinal distance. At rest, the residual slope was found to be 0.0015.

**Table 1:** Coordinates of the corners at each lateral window of the flume.

		Left down corner	Left up corner	Right down corner	Right up corner
**Window 1**	*x* (m)	0.055	0.055	1.843	1.843
	*z* (m)	0.025	0.97	0.025	0.97
**Window 2**	*x* (m)	1.903	1.903	3.691	3.691
	*z* (m)	0.025	0.97	0.025	0.97
**Window 3**	*x* (m)	3.806	3.806	5.594	5.594
	*z* (m)	0.025	0.970	0.025	0.97
**Window 4**	*x* (m)	5.654	5.654	7.442	7.442
	*z* (m)	0.025	0.97	0.025	0.97
**Window 5**	*x* (m)	7.557	7.557	9.345	9.345
	*z* (m)	0.025	0.97	0.025	0.97
**Window 6**	*x* (m)	9.405	9.405	11.193	11.193
	*z* (m)	0.025	0.97	0.025	0.97
**Window 7**	*x* (m)	11.308	11.308	13.096	13.096
	*z* (m)	0.025	0.97	0.025	0.97
**Window 8**	*x* (m)	13.156	13.156	14.944	14.944
	*z* (m)	0.025	0.97	0.025	0.97

**Fig. 3:**
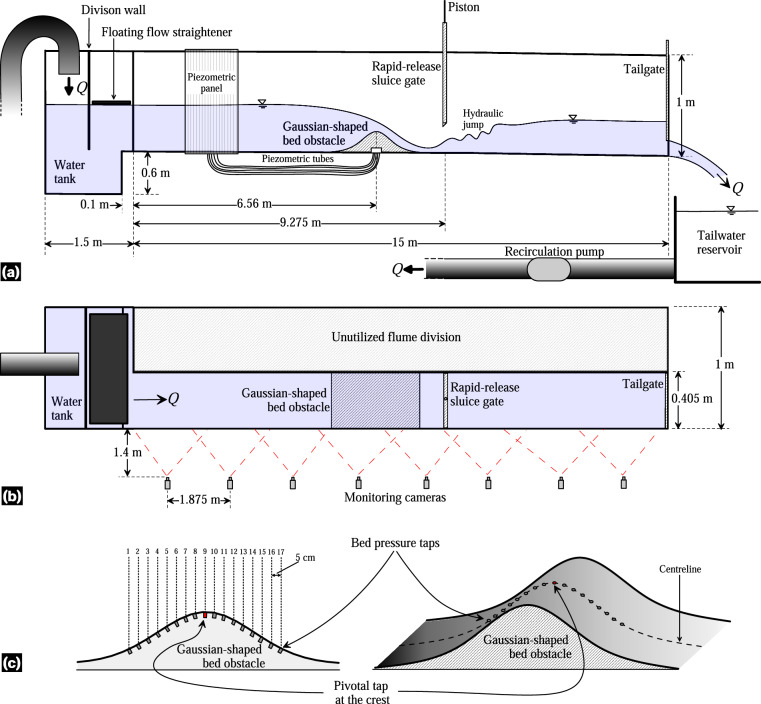
Sketch of the open-channel flume: (**a**) side view, (**b**) plan view, and (**c**) Gaussian-shaped bed obstacle with bed pressure taps distribution.

The flow discharge in the flume was supplied through two different systems. The first system involved a unidirectional setup of pumps and headwater reservoirs (Fig. 1d). The second system was a recirculating system that utilized the tailwater reservoir (Fig. 1e). For the steady hydraulic jump experiments conducted in this study, the second system was used to maintain a constant discharge over time. A single centrifugal pump (WA Motors 1L160M-4 B35 PTC) with an adjustable frequency drive (AFD) was used to recirculate the flow. It had a maximum discharge capacity of *Q*_*max*_ = 0.078 m^3^/s, resulting in a theoretical maximum unit discharge of *q*_*max*_ = 0.1926 m^2^/s. To accurately measure the discharge *Q*, a SIEMENS SITRANS F M MAG/5000 flowmeter was installed in the recirculating pipe. The flowmeter offered a measurement accuracy of ±0.4%. Several components were integrated into the flume setup, including an upstream water tank with a division wall and a floating flow straightener to ensure stabilized flow at the inlet. Note that the inflow tank was not divided, as is typically done with the nominal width reduction in the flume. This design choice results in the displacement of the inlet flume section to one side of the inflow tank (Fig. 3b). As shown in Fig. 3a,b, the tank’s geometric characteristics are described as a box measuring 1.5 × 1.6 × 1 m, with a step of 0.1 m at 0.6 m from its bottom. The tank features a division wall to dissipate energy and a floating plate element as a flow straightener. These elements were strategically installed to minimize the asymmetric inflow conditions arising from the asymmetrical geometry at the flume inlet. A high-speed release sluice gate, driven by a pneumatic system, was positioned in the flume to generate unsteady dam-break-like water waves (Fig. 2a). Additionally, a tailgate with mechanical action was installed to control the flow depth at the outlet (Fig. 2b). The rapid sluice gate only affected the reduced section of the flume and was located at *x* = 9.275 m from the flume inlet (Fig. 3a,b). It was manually operated with a remote control, allowing for a fast opening of 0.5 m in 0.162 s under a pneumatic system pressure of 9 bar. According to Lauber and Hager^25^, the dimensionless time *T* [ = *t*_0_(*h*_0_/*g*)^1/2^] for a dam-break gate lift must be below $\sqrt{2}$. Here, *t*_0_ is the physically-achieved gate opening time, *h*_0_ is the initial height of the impounded water, and *g* is the gravitational acceleration. For *h*_0  _= 0.5 m, *t*_0_ turns out to be 0.3193 s. The movement of the dam-break gate was characterized with a high-speed camera FASTEC TS5 able at capturing up to 253 frames-per-second, which leads to a time gap between recorded instants of 0.004 s approximately. This indicates that the rapid sluice gate opening is well-suited for instantaneous dam-break like experiments.

The Gaussian hump, installed as an instream obstacle being centred at *x*_*crest*_ = 6.56 m (Figs. 2, 3a), is described by the following equation:

2 $${z}_{obs}=0.209\,\exp \left[-\frac{1}{2}{\left(\frac{x-{x}_{crest}}{0.254}\right)}^{2}\right]$$

where *z*_*obs*_ is the local obstacle height above the flume bed and *x*_*crest*_ is the location of the obstacle crest. Note that Eq. (2) represents the outcome of adjusting the constructed Gaussian obstacle to a Gaussian function. The Gaussian shape was originally designed as *z*_*b*_ = 0.2 exp[−(1/2)((*x* − *x*_*crest*_)/0.24)^2^]. Following the construction and installation of the bed-form in the flume manufacturing errors occurred, and the observed Gaussian obstacle surface underwent digitalization and fine-tuning to match a Gaussian function, resulting in Eq. (2) with an excellent fit (*R*^2^ = 0.998). The obstacle was constructed using stainless steel, with an empty interior to accommodate the installation of piezometric tubes for bed pressure measurements (Fig. 2c). This construction resulted in two different surfaces (in terms of roughness) in the flume: (i) the stainless-steel bed obstacle and (ii) the flume floor having an autolevelling two-component polyurethane coating (MasterTop BC 375 N by Master Builders Solutions). A total of 17 piezometric tapings were strategically positioned along the centreline of the obstacle. These tapings extended to a piezometric panel attached to the sidewall of the flume, allowing for the bed pressure measurements at the obstacle centreline (Fig. 2d).

### Tests procedure

The experimental campaign consisted of two types of experiments: steady and unsteady flow experiments. Both types of experiments aimed at to observe and analyse the behaviour of hydraulic jumps in interaction with both the instream obstacle and the flume bed configuration.

#### Steady hydraulic jump experiments

**Fig. 4:**
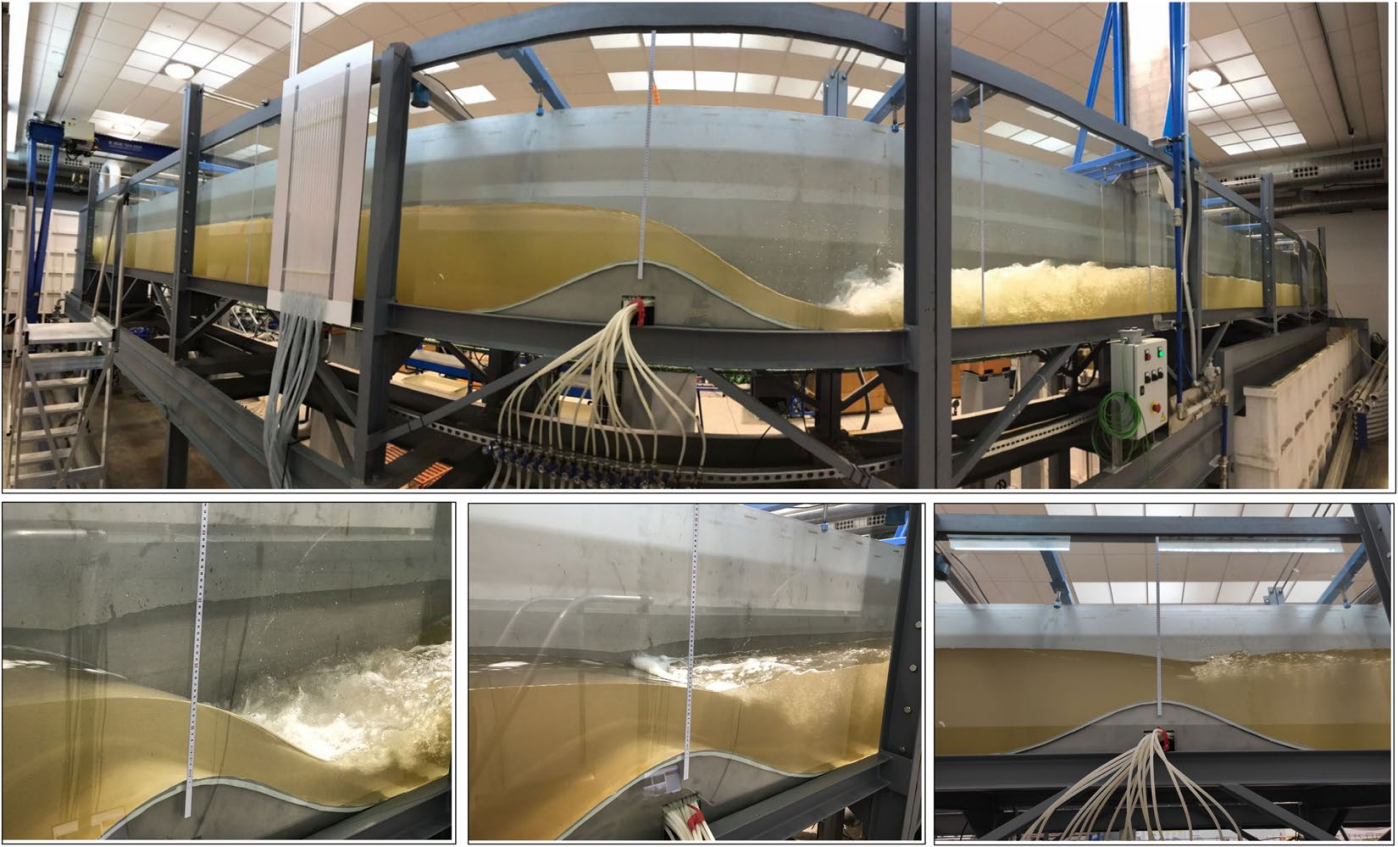
Photographs of the steady hydraulic jump experiments.

A total of eight steady hydraulic jump experiments were conducted, with two different sets of experiments carried out at different times. All eight experiments followed the same test procedure and data production methodology. However, for two of the experiments (experiments 7 and 8 in Table 2), the piezometric device was not available. Thus, only the bed pressure readings were available for the remaining six experiments. In the first six experiments, the maximum discharge was utilized, whereas for experiments 7 and 8, a fraction of the maximum discharge was released by adjusting the pump using the AFD. The desired position of the hydraulic jump roller was achieved by gradually adjusting the tailgate opening, thereby modifying the flow conditions downstream of the bed obstacle crest. This resulted in a total of 8 different steady experiments. These experiments of steady hydraulic jumps aimed at to explore the different submergence states of the leeside of the Gaussian-shaped bed obstacle, ranging from fully submerged to partially submerged configurations, including the unsubmerged states (i.e., jump located downstream of the leeside). Note that the term ‘submerged’ refers to the position of the hydraulic jump, while the Gaussian-shaped bed obstacle remained constantly submerged throughout the experiments. During each experiment, the discharge was measured using a flowmeter. The flow and boundary configurations were then maintained for a duration of 30 min to ensure the flow steadiness, monitored by observing the position of the starting point of the roller. Thereafter, a short 7-second video was recorded for each window from the third to the eighth windows. These videos were later used to extract frames for the experimental data during the postprocessing stage. Table 2 provides details of the flow and boundary conditions for all the steady flow experiments, including the discharge per unit width *q* for convenience. It is worth noting that, even for the same full AFD conditions, the discharge varied due to the changing conditions in the tailwater reservoir between tests. Figure 4 presents the general photographs illustrating the execution of this set of experiments.

**Table 2:** Flow and boundary conditions details for steady hydraulic jump experiments.

Experiment	AFD (%)	*Q* (m^3^/s)	*q* (m^2^/s)	Tailgate opening (m)
1	100	0.0741	0.1831	0.139
2	100	0.0764	0.1886	0.132
3	100	0.0765	0.1888	0.126
4	100	0.0767	0.1893	0.12
5	100	0.0746	0.1841	0.114
6	100	0.07428	0.1834	0.144
7	60	0.05394	0.1332	0.113
8	20	0.015	0.037	0.052

#### Unsteady hydraulic jump experiments

**Fig. 5:**
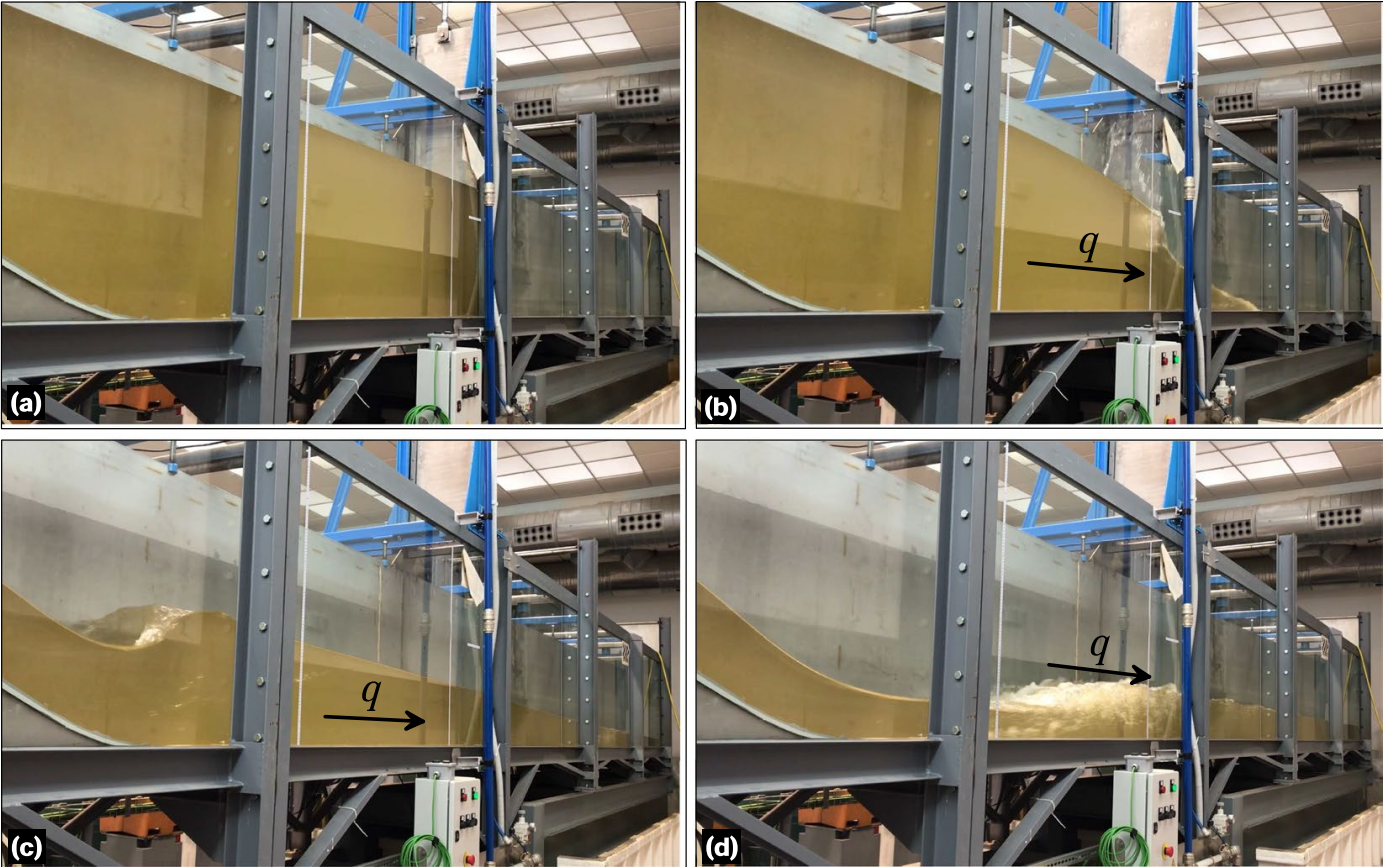
Photographs of the unsteady hydraulic jump experiment 1 with open tailgate: (**a**) *t* = 0, (**b**) *t *≈ 0.5 s, (**c**) *t* ≈ 3 s, and (**d**) *t* ≈ 7 s.

**Fig. 6:**
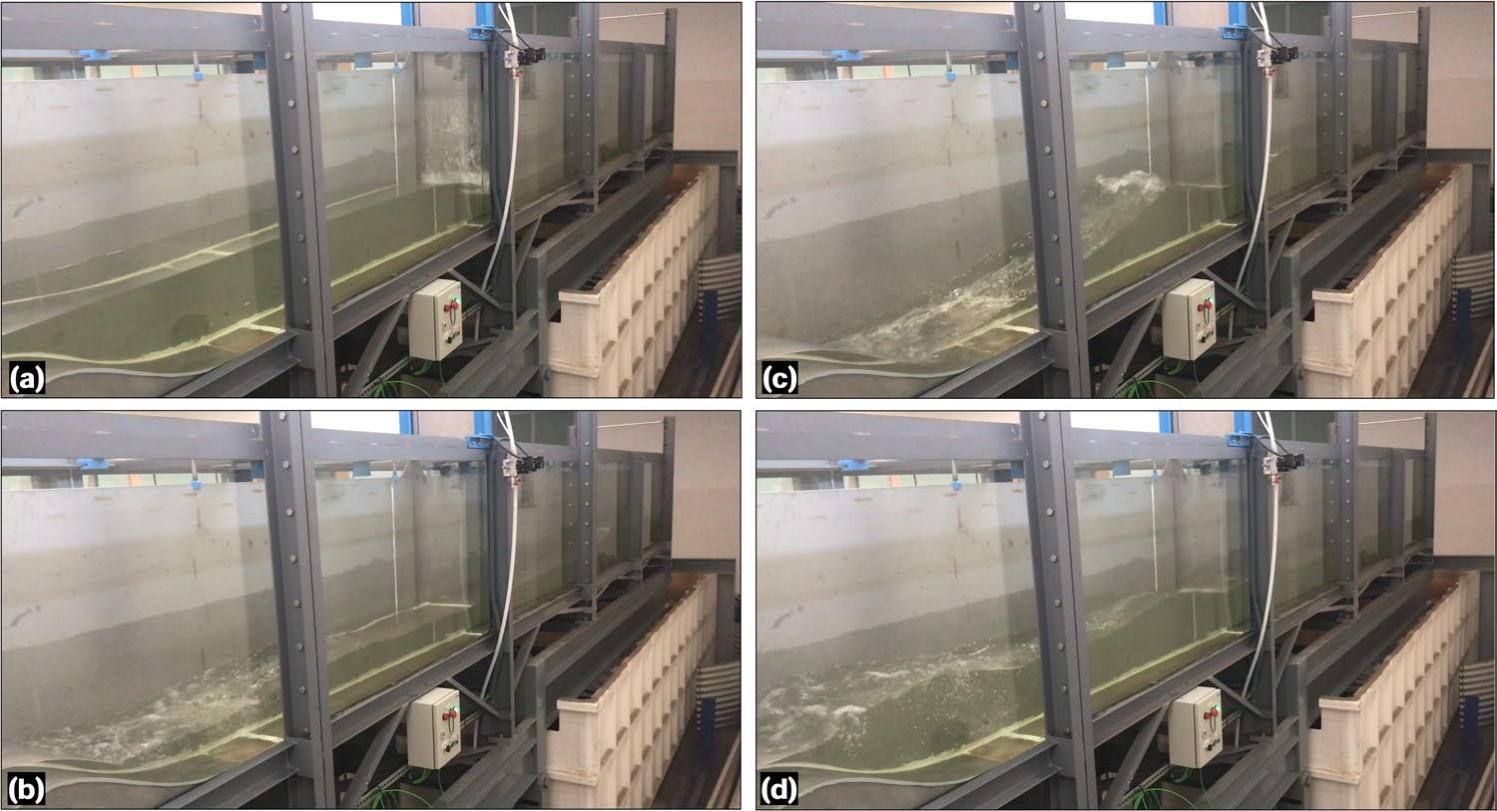
Photographs of the unsteady hydraulic jump experiment 7 with closed tailgate: (**a**) *t* = 0.2 s, (**b**) *t* ≈ 5 s, (**c**) *t* ≈ 8 s, and (**d**) *t* ≈ 12 s.

Eight experiments were conducted to characterize unsteady hydraulic jumps under the flume and obstacle configuration. These experiments focused on analysing the rapid formation and development of hydraulic jumps by using a rapid-release sluice gate as well as the transformation of waves over the Gaussian obstacle. The first three experiments were conducted free of reflective waves by setting the tailgate fully opened, while the five remaining experiments were conducted accounting for reflective waves by keeping the tailgate closed (Table 3). The flow discharge at the inlet boundary was set to zero through all the unsteady tests. Using the rapid-release sluice gate with different upstream flow depths, dam-break-like waves were generated, which then evolved into the formation and development of hydraulic jumps in interaction with the obstacle (Fig. 5) and, eventually, with reflected waves (experiments 4 to 8 in Table 3) (Fig. 6). The front of the hydraulic jump progressively evolved between the Gaussian-shaped bed and the flume end. The execution sequence for each experiment was as follows: (i) the tailgate was adjusted according to the boundary condition in each experiment (Table 3) and the sluice gate was closed, (ii) the recirculating pump was activated at the minimum AFD of 20% to slowly fill the upstream flume portion, ensuring no traveling waves were generated until reaching the desired water level, while the downstream flume portion was flooded using a hose when required, and (iii) after 10 min of rest to ensure a stable water surface, the sluice gate was released to initiate the flow. Table 3 provides details of the initial conditions and boundaries for the unsteady hydraulic jump experiments. The initial flow depth upstream of the sluice gate was measured at *x* = 8.962 m as a reference point, while the downstream one was read at *x* = 9.731 m. Using the monitoring camera system, the entire process was recorded for at least 12 s from the release of the sluice gate to capture the key hydrodynamics in both the experiments with and without reflective waves. Thus, in experiments 1 to 3, the films extended until the moving hydraulic jump exits the flume, while they do until the reflective wave trains reach the first upstream wetted section in experiments 4 to 8 (note that this section is on the Gaussian-obstacle for experiment 4).

**Table 3:** Flow and boundary details for unsteady hydraulic jump experiments.

Experiment	Upstream flow depth (m)	Downstream flow depth (m)	Tailgate opening (m)
1	0.65	Dry	1
2	0.531	Dry	1
3	0.435	Dry	1
4	0.209	Dry	0
5	0.295	Dry	0
6	0.301	0.116	0
7	0.313	0.189	0
8	0.306	0.241	0

**Fig. 7:**
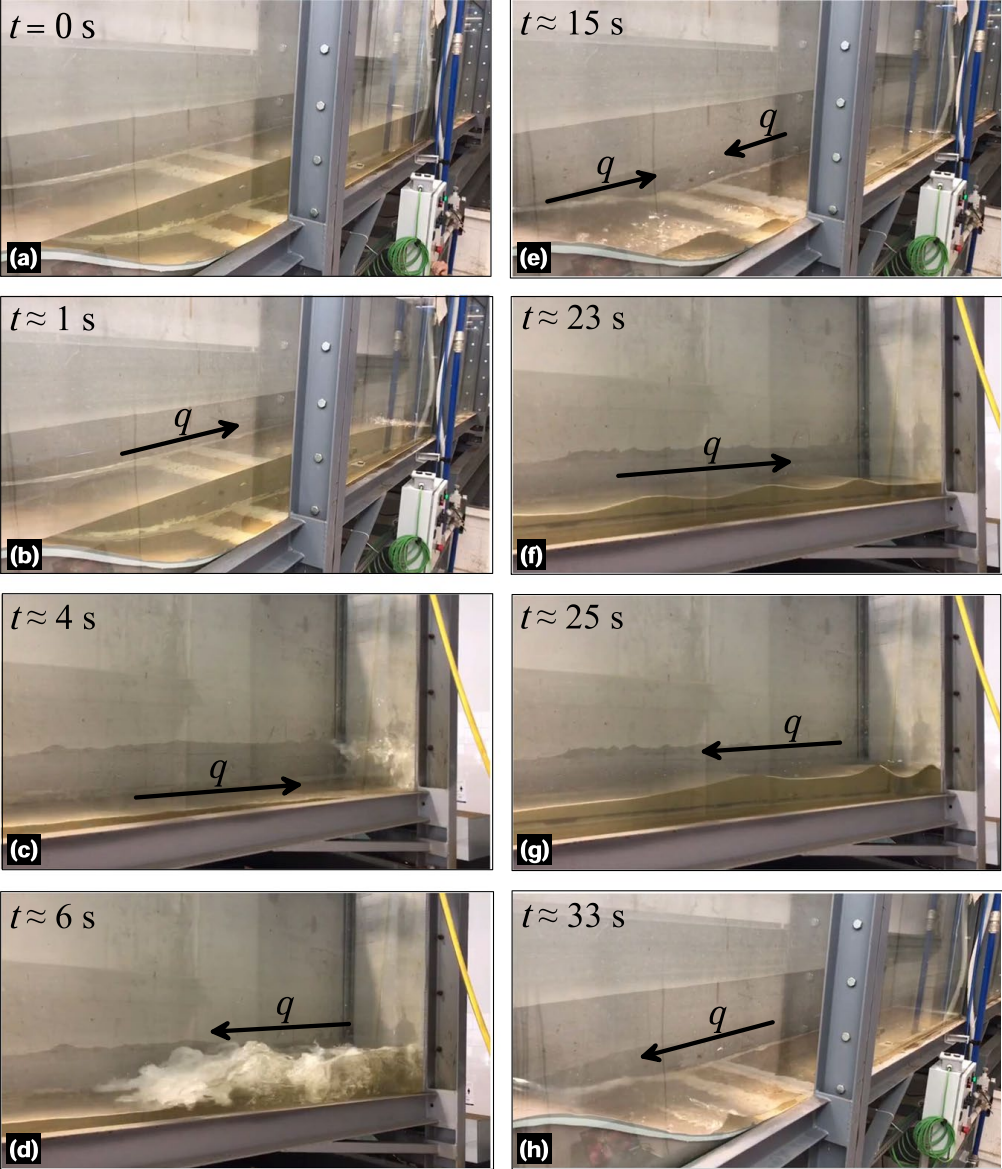
Photographs of the unsteady hydraulic jump experiment 4 with closed tailgate at various time instants: panels (**a**–**d**) correspond to the initial bore and waves reflexion and panels (**e**–**h**) correspond to the second train of waves.

The unsteady experiment 4 (Table 3) is unique in the series as aiming at describing the transformation, breaking, and the run-up of waves over the Gaussian-shaped bed profile, where unsteady hydraulic jumps are formed (Fig. 7). The results of this experiment have relevance not only for hydraulic engineering but also for other disciplines, such as ocean and coastal engineering, thus indicating their potential utilization for the scientific community. To conduct this experiment, the tailgate was closed and only the upstream portion of the flume between the obstacle and the sluice gate was filled with water (Fig. 7). The flow depth in the upper part of the flume, from the inlet to the obstacle, was intentionally kept below the obstacle crest. This was done to prevent mass interchange between both sides of the obstacle throughout the experiment. The experiment was repeated several times to ensure that the reflected train of waves did not exceed the obstacle crest, thereby maintaining the initial water mass throughout the experiment. The sequence of the experiment was as follows: (i) the sluice gate and the tailgate were closed, and the flume was inclined to its maximum position, (ii) the recirculating pump was activated at the minimum AFD to fill the portion between the Gaussian-shaped bed profile and the sluice gate, (iii) the flume inclination was then removed to maintain the flow depth between the obstacle and the sluice gate at the level of the obstacle crest (as a consequence, the upper part of the flume was filled with the remaining water mass), (iv) after 10 min of rest to ensure a stable water surface, the sluice gate was released to initiate the flow. The dam-break flow evolved, moving towards the closed tailgate, and subsequently resulted in a reflected train of waves that travelled back to the obstacle, causing a run-up (Fig. 7). This process was recorded using the monitoring camera system, capturing two complete run-ups of the train of waves. This suggests that the dam-break waves were reflected twice by the flume end wall and reached the obstacle twice.

### Free surface level measurements

The measurement and characterization of the free surface of flow during the experiments involved a multi-step process that included image acquisition, image postprocessing and data production from images. Instead of capturing snapshots at different test instants, short videos of the experiments were recorded. These videos were later used to extract images (frames), which were postprocessed to obtain the data.

#### Image acquisition process

**Fig. 8:**
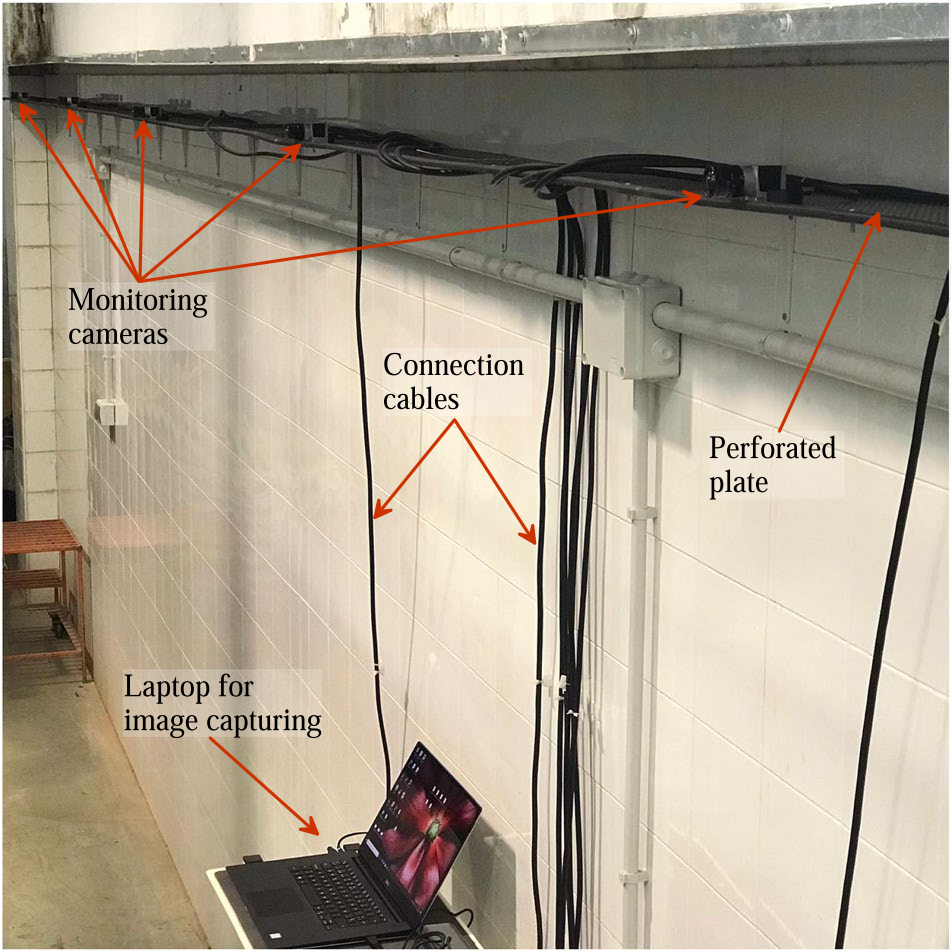
Monitoring cameras and their connection to the central laptop.

For image acquisition, a synchronized camera monitoring system was used. The monitoring system consisted of 8 Basler Ace acA1920-40uc cameras, each equipped with a 6 mm focal length lens. These cameras had a maximum resolution of 1920 × 1200 pixels, with 96 ppi, and allowed operation at 40 frames/s (fps) maximum. Considering the window dimensions given in Table 1, at this resolution, the pixel width was approximately 0.00132 m. This value defines the maximum accuracy achievable in the digitalization process. The cameras were positioned perpendicular to each lateral window of the flume, maintaining an interdistance of 1.875 m (Fig. 3). Cameras were installed 1.4 m away from the flume wall, ensuring that the entire window vision was captured adequately due to operational angle of the focal length lens. To allow for adjustment of the camera positions, they were mounted on a perforated plate along the laboratory wall. The cameras were interconnected with a special wire for synchronization and subsequently connected individually to a laptop for the image acquisition process (Fig. 8). While the Balser Pylon software facilitated the activation of camera drivers, settings, and synchronization, the Streams7 software was used to configure the cameras optimally and to record the videos. To avoid blinking issues that occurred when all 8 cameras were operated simultaneously at high frame rates (over 35 fps), the cameras were set to operate at 25 fps. This frame rate was sufficient for studying test instants below 1 s, with a time interval of 0.04 s between frames. Note that although this work did not analyse time instants below 0.04 s, the camera monitoring system used allowed for examination of such instants by increasing the fps setting during video recording. Once all the cameras were confirmed to be operating at the desired conditions, the videos were recorded simultaneously using the Streams7 software. The recording sequence involved initiating the recoding first and then conducting the experiments. The duration of the recorded videos varied depending on the specific experiment, allowing to capture the important flow physics. For instance, the durations were approximately 7 s for steady flow experiments, 20 s for unsteady flow experiments, and 40 s for the run-up test (experiment 4 in Table 3).

#### Image postprocessing

**Fig. 9:**
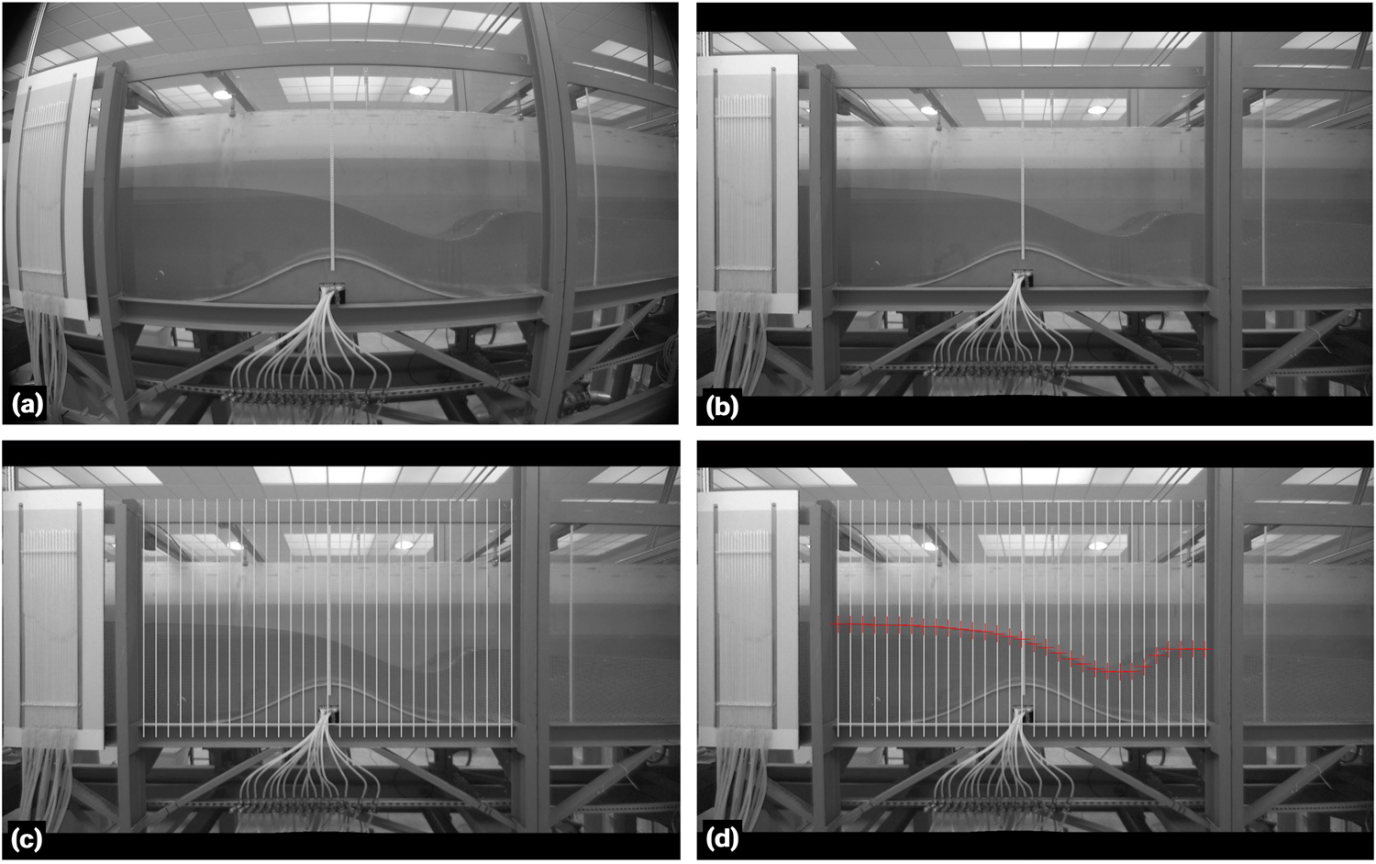
Sequence of the image distortion correction and data digitalization: (**a**) frame extraction, (**b**) image correction, (**c**) vertical grid superposition, and (**d**) free surface flow digitalization.

The image postprocessing involved two main steps: frame extraction at the desired time instant and image distortion correction. To extract frames from the recorded videos, a custom algorithm was programmed in MATLAB. The algorithm read each video and extracted frames at a specified time step. Table 4 provides the algorithm for the frame extraction. The frame extraction time instants varied depending on the type of experiment conducted. For the steady experiments, six time instants were selected with a time step of Δ*t* = 1 s to produce instantaneous data profiles. The extracted frames were later averaged over time interval *t* ∈ [0, 5] s with Δ*t* = 1 s to obtain additional results. For the unsteady experiments 1−3 and 5−8 (Table 3), frames were extracted at time instants *t* ∈ [0, 16] s with Δ*t* = 1 s. For the run-up test (experiment 4 in Table 3), frames were extracted at *t* = 0, 1, 3.12, 3.64, 5.32, 13.6, 14.04, 14.36, 15.68, 22.92, 23.52, 33.32, 33.56, 33.96, 34.48 s to capture the important flow phenomena of the experiment accurately. After frame extraction, image distortion induced by the use of the 6 mm focal length lens was corrected. The Panorama Corrector 2.2 by Altostorm was employed for this purpose instead of a custom algorithm, as we did not find a suitable and efficient method for developing such code. The lateral and top/bottom steel beams of each window framework were used as reference vertical and horizontal lines, respectively, in the pseudo-panorama of each video frame processed. This correction ensured that the window area, framed by the beams of perfect verticality and horizontality, was free from image aberration (Fig. 9a,b). The image correction permitted us to digitalize the data subsequently minimizing distortion errors. The digitalization of the free surface of flow was conducted manually, as alternative edge-detection algorithms and binarization were implemented with unsatisfactory results, primarily due to the light conditions of the images. Although the manual process was tedious, it proved to be suitable for achieving precision in the image postprocessing (with a maximum accuracy of ±0.00132 m by pixel width as noted in previous sections). To facilitate the digitalization process, additional adjustments were made to improve image quality, including contrast and brightness adjustments for each image, using a commercial software regardless of any specific method.

**Table 4:** Algorithm for the frame extraction.

#### Data production

**Fig. 10:**
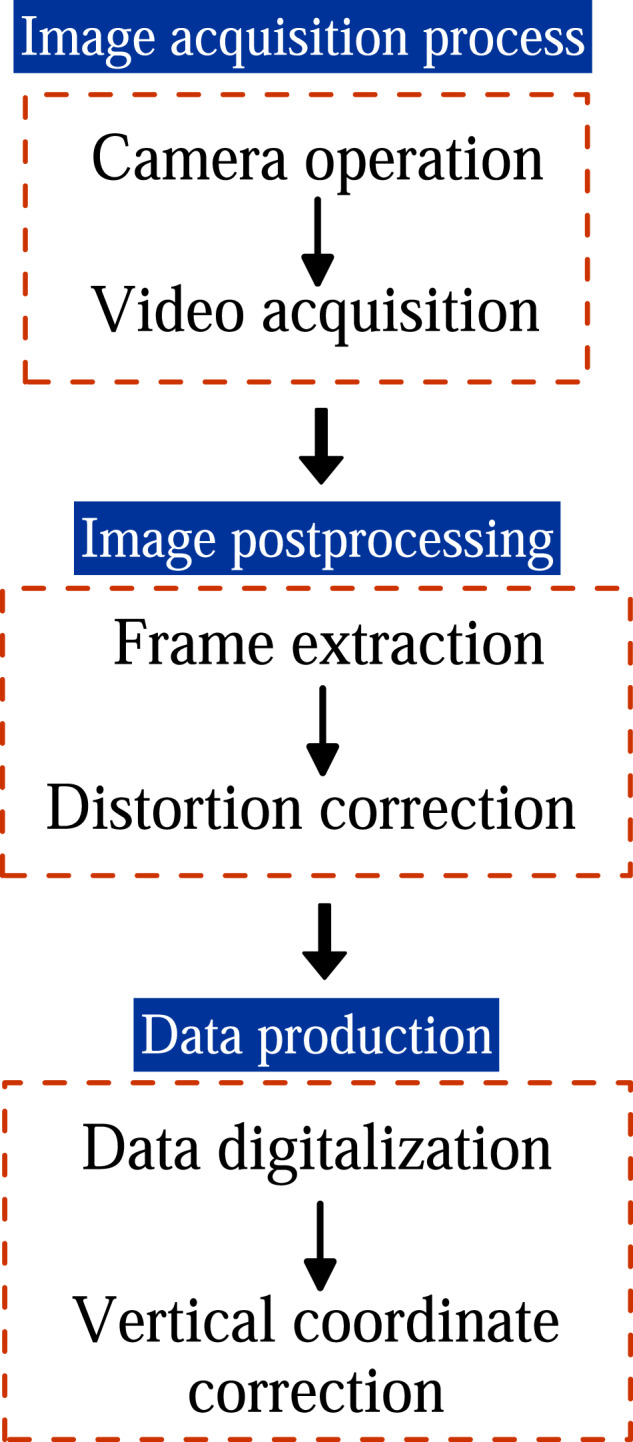
Workflow diagram for measuring the free surface level.

After correcting any image distortion and enhancing their quality, the extracted frames at the desired time instants were used to digitalize the free surface of the flow. This was done using the Grapher 19.2.305 software by Golden Software LLC. The digitalization processed entailed the following steps: (i) importing a corrected frame into the software, (ii) specifying the coordinates of the image by defining a set of two points on the plane, (iii) placing a grid of vertical divisions on the image (Fig. 9c), and (iv) digitalizing the intersections of the grid divisions with the free surface line (Fig. 9d). The grid was introduced to maintain consistent data density and interdistance in each flume window of evaluation. Note that the intersections, indicating the data points for extraction, were visually detected, relying on the accuracy determined by pixel length in the images. Another potential source of error is the visual crystal window effect, which varies from zero near the perpendicular camera-window intersection to a maximum near the flume bed. The comparison with the data extracted by using the point gauge for technical validation was excellent, indicating that this source of error may be negligible within the framework of this study. After experimenting with different grid sizes, a 30-division grid was chosen as the optimum option for characterizing all important hydraulic processes, resulting in 29 digitalized points per flume window/image. The coordinates for each flume window were obtained using the corner coordinates listed in Table 1. Thereby, the resulting digitalized data were referenced to a virtual horizontal plane representing the flume bed. At the flume locations where double beams serve as vertical steel braces, no free surface data will appear in the database. This occurs approximately at *x* = 3.69, 7.44, and 11.19 m (Figs. 1, 2, 4, 5). Continuity in the water level data is maintained in the remaining portions, as the grid spacing and the single beam joint width are of the same order, such as 0.0625 and 0.06 m, respectively. To account for the flume bed bathymetry, the vertical coordinate of data needed rectification. Table 5 shows the algorithm used for translating the vertical coordinate into the global system of coordinates, taking into account the cantilever Eq. (1) and the flume residual slope. This rectification process was previously followed in Gamero *et al*.^23^ for the same flume setup and different experiments, resulting in an accurate matching between flow model simulations and post-processed data, providing additional support for neglecting the source of error introduced by the crystal window effect in this study. Figure 10 illustrates the entire process of measuring the free surface level data through a workflow diagram.

**Table 5:** Algorithm for the frame extraction.

### Pressure measurements in steady flow

**Fig. 11:**
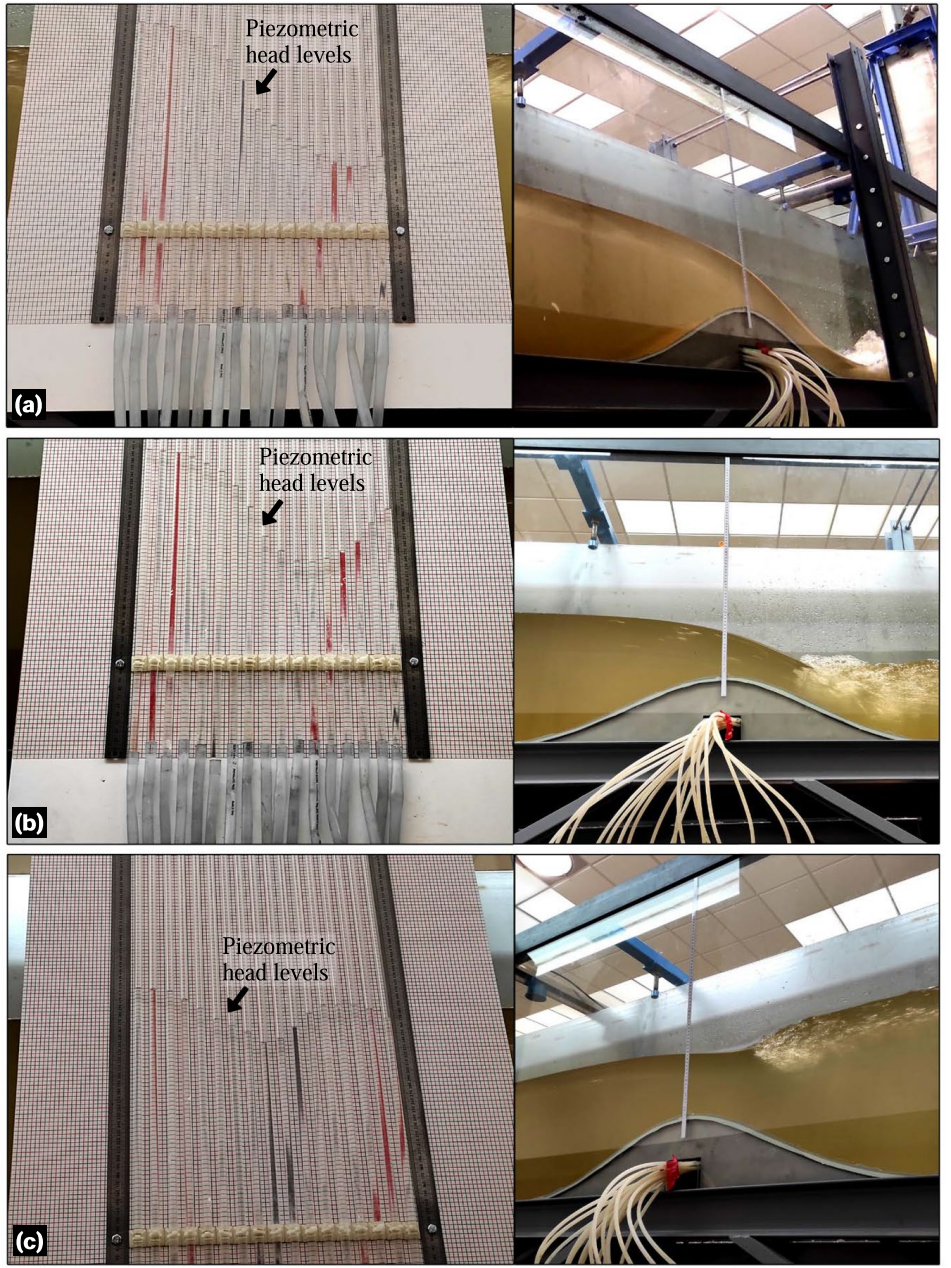
Piezometric head levels for steady hydraulic jumps corresponding to (**a**) experiment 1, (**b**) experiment 2 and (**c**) experiment 3.

For the steady hydraulic jump experiments 1−6 (Table 2), the bed pressure was measured using piezometers installed in the Gaussian-shaped bed profile. The pressure taps were installed equidistant along the centreline of the Gaussian profile, with an interdistance of 0.05 m and a pivotal element at the very crest of the shape (Fig. 3c). Note that no more pressure taps were available at the flume, because no piezometers were installed in the plain flume bed for this facility at UCO. The piezometers had a reaction time (i.e., not instantaneous) to adjust to pressure head variations. This is why the piezometers were not used during the unsteady experiments. Therefore, bed pressure readings were taken after a steady time of 30 min to ensure flow steadiness for the experiments listed in Table 2. Prior to conducting the steady experiments, the reference level (zero pressure head) in the piezometric tubes was calibrated. To do this, the flume was initially filled with still water up to a 50 cm head, providing enough hydrostatic pressure to drain and eliminate bubbles in the tubes, thereby ensuring fluid pressure continuity along the tubes. Then, the flume was slowly drained to fill the piezometers with water up to the very surface. The water level in the piezometric panel was noted as the reference level. By subtracting the piezometric level measured during the experiments from the reference level, the piezometric head was calculated. Pressure head measurements were conducted manually *in-situ* using a calibrated steel ruler and a millimetre board placed behind the tubes, which served as the background for the piezometric panel. The estimated error for the measurements was ±0.5 mm due to a combination of several factors, namely the water meniscus, static water level fluctuation, and eye precision. Figure 11 shows the piezometric panel operating under steady conditions for experiments 1, 2, and 3.

## Data Records

Two data packages are available a ZENODO repository publication^24^ for this work. The main data package includes free surface measurements, corresponding bed levels, and bed pressure measurements along the Gaussian-shaped bed profile. Additionally, the secondary data package provides individual corrected images, panoramas composed of the corrected images for each analysed time instant in the experiments, and a representation of the free surface data. Table 6 summarizes the available data packages in the ZENODO repository publication^24^ and their contents. A detailed description of each data package is provided below.

**Table 6:** Data packages files and contents in^24^.

Data package	Data file name	Does it contain free surface and bed level data?	Does it contain bed pressure data along the Gaussian-shaped bed profile?
Measured data	SCIDATA2024_SteadyExps_1_to_8_data.xlsx	Yes	Yes (for experiments 1−6)
	SCIDATA2024_UnsteadyExps_1_to_8_data.xlsx	Yes	No
Images and plots	SCIDATA2024_SteadyExps_1_to_8_img.zip	—	—
	SCIDATA2024_UnsteadyExps_1_to_8_img.zip	—	—

### Free surface, bed level, and pressure head experimental data

This main data package in^24^ is organized into two files: (i) a data file for the steady hydraulic jump experiments listed in Table 2 and (ii) a data file for the unsteady hydraulic jump experiments listed in Table 3. In the first file (SCIDATA2024_SteadyExps_1_to_8_data.xlsx), the bed pressure readings along the Gaussian-shaped bed profile are provided for the steady experiments 1−6, as mentioned in the preceding sections. For each test in the steady experiments set, this file includes the digitalized data at each of the six-time instants, as well as the final time-averaged data. Although *z*_*b*_ (bed level) can be implemented analytically using Eq. (2), it was measured and provided at every *x*-coordinate for the *z*_*s*_ (free surface level) data to facilitate operation and dissemination. The vertical coordinate was only corrected in the final time-average data of each steady test. It is worth noting that the final time-averaged data for each steady experiment consists of two vectors [*x*, *z*_*b*_, *z*_*s*_], which are ready to be used by modellers. On the other hand, the data without vertical coordinate correction (directly extracted from images) allows modellers to apply their own correcting algorithms if desired. The second file (SCIDATA2024_UnsteadyExps_1_to_8_data.xlsx) includes the vertically corrected free surface and bed level data of the unsteady experiments 1–8 in Table 3 by using the algorithm given in Table 5. The data are provided in Excel files in^24^, with each experiment organized into sheets.

### Corrected panoramas of experimental images

**Fig. 12:**
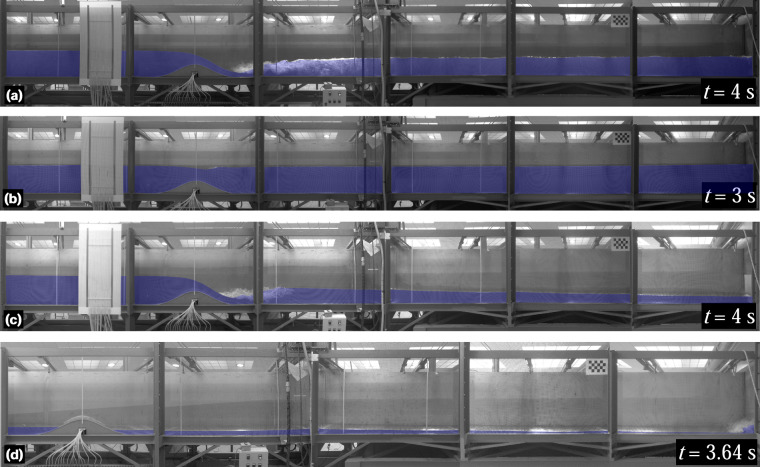
Panoramas for the (**a**) steady experiment 1, (**b**) steady experiment 4, (**c**) unsteady experiment 2, and (**d**) unsteady experiment 4.

**Fig. 13:**
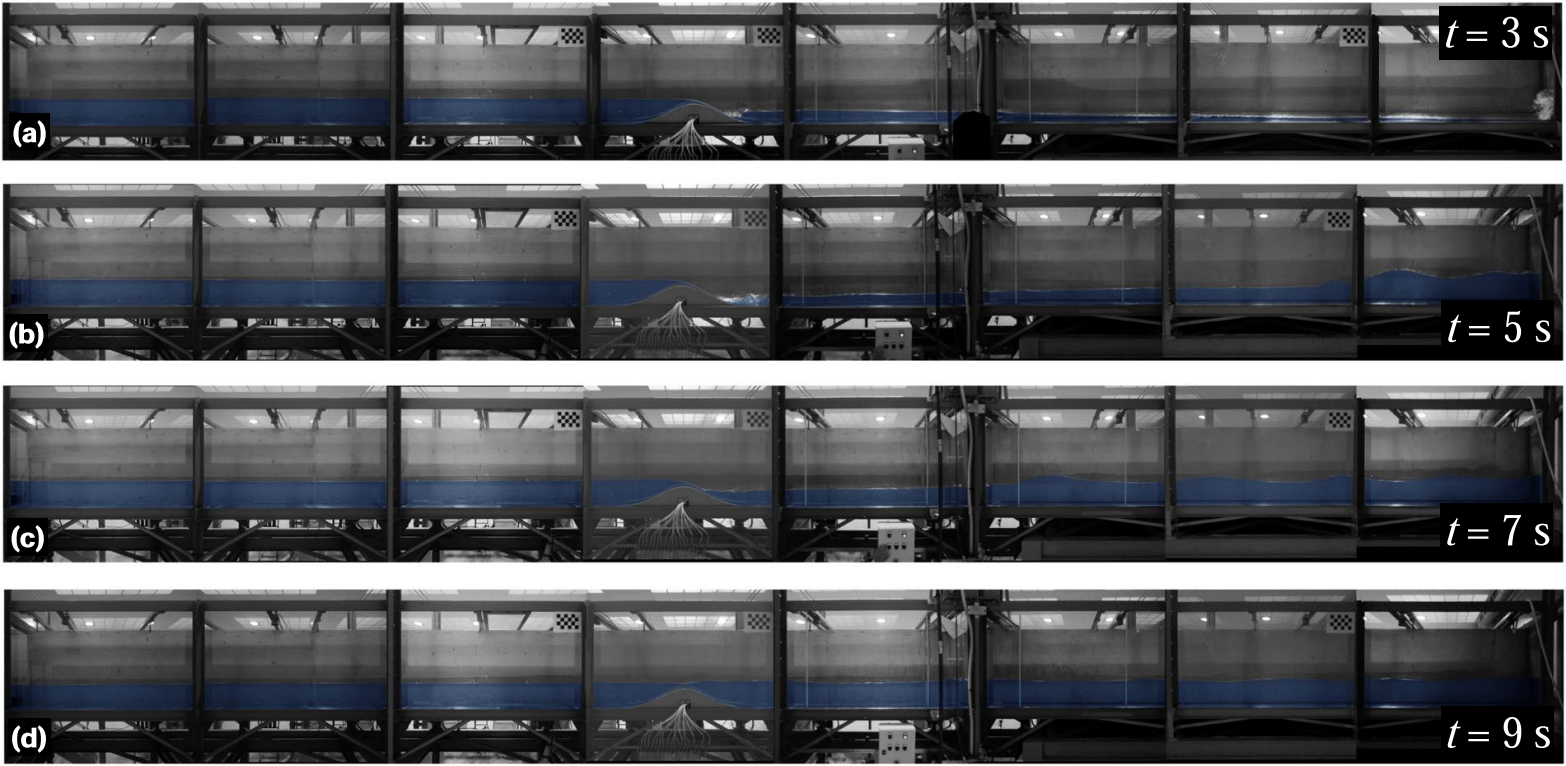
Panoramas for the unsteady experiments (**a**) 5, (**b**) 6, (**c**) 7, and (**d**) unsteady 8.

The second data package in^24^ is organized into two compressed files, corresponding to the steady and unsteady hydraulic jump experiments, respectively. These files contain high resolution images that underwent lens distortion correction for all time instants and cameras in each experiment. In addition, panoramas of all assessed time instants, created manually assembling the corrected images from the cameras involved, are provided for each test. The panoramas serve as a means for scientists and readers to quickly evaluate the flow phenomena generated in each test at a specific time instant. However, it is discouraged to use the panoramas for data digitalization. For accurate digitalization, the isolated corrected images are the recommended choice. Figure 12 displays some panoramas for the steady experiments 1 and 4 (Fig. 12a,b), as well as for the unsteady experiments 2 and 4 (Fig. 12c,d). Figure 13 shows several panoramas for the unsteady experiments 5−8. Note that, in^24^, panoramas examples were arranged in two figures because of the number of cameras involved in the different experiments.

## Technical Validation

### Free surface data

**Fig. 14:**
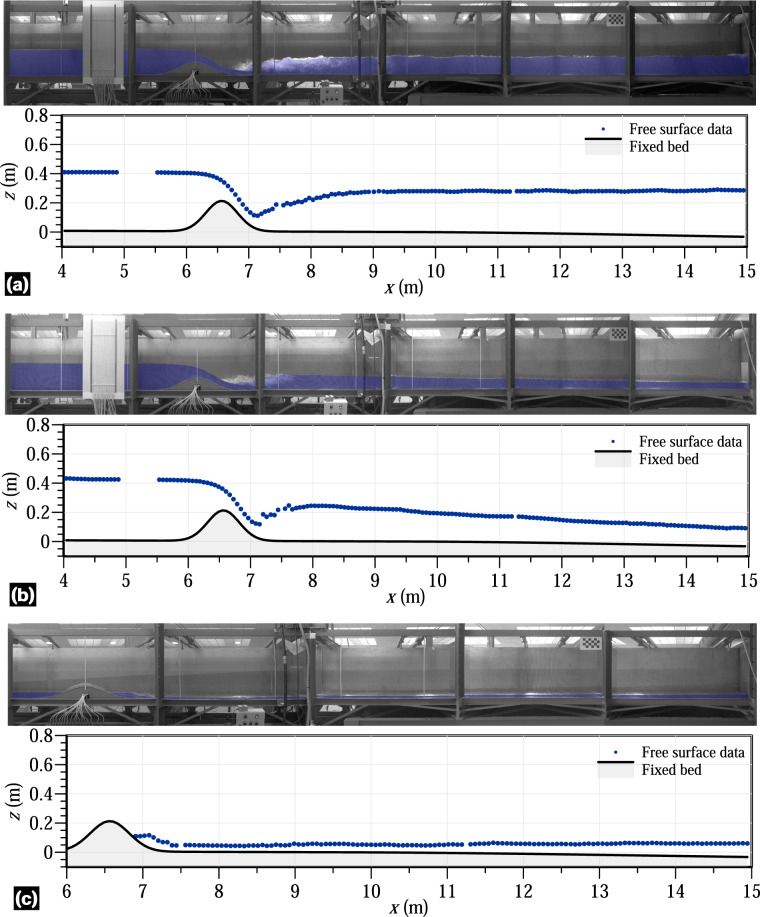
Free surface data and bed level for (**a**) steady experiment 1, (**b**) unsteady experiment 2 at *t* = 4 s, and (**c**) unsteady experiment 4 at *t* = 14.36 s.

**Fig. 15:**
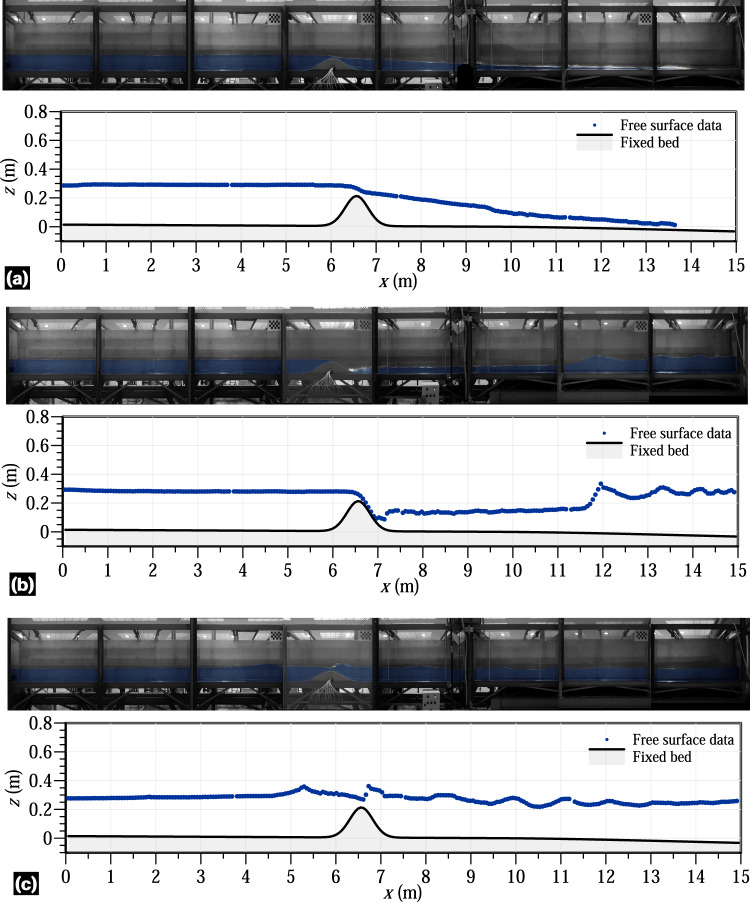
Free surface data and bed level for the unsteady experiments (**a**) 5 at *t* = 2 s, (**b**) 6 at *t* = 6 s, and (**c**) 7 at *t* = 10 s.

Steady and unsteady flow depth variables were measured using a multi-process involving image acquisition, postprocessing and data digitalization, as explained in the method section. Point gauges with ±0.1 mm accuracy were manually used at different positions during the steady experiments to evaluate the uncertainty of the digitalized free surface readings. Notable free surface fluctuations were observed in the subcritical flow portions of the flume, especially after the hydraulic jumps. Therefore, this validation technique was directly applied upstream of the bed obstacle and over the obstacle, whereas the averaged values were used downstream of the jumps in the steady experiments. Note that this validation technique could not be applied to the unsteady experiments. The comparison between the digitalized data and the measured data by point gauges showed an almost perfect correlation, with determination coefficients (*R*^2^) over 0.98. To extend the validity of digitalization to the unsteady experiments, the positions of the cameras remained unchanged throughout the entire laboratory campaign. Additionally, 36 experiments involving steady flow over the flume obstacle without hydraulic jumps downstream were performed and processed to further validate the method for measuring free surface levels through digitalization^23^. These experiments lasted at least 30 min to ensure the flow steadiness. The free surface of flow was measured using a point gauge and 8 SIEMENS ECHOMAX XRS-5 ultrasonic transducers, which were previously calibrated through still flow experiments in the flume. The accuracy of the ultrasonic readings was ±0.2 mm, whereas the accuracy of the point gauges measurements was ±0.1 mm. However, the ultrasonic probes could not be used in flow portions with a significant free surface curvature due to the diverging directions of the return ultrasonic waves caused by their narrow beam angle of 10° within the detection cone. This limitation restricted the application of these sensors in flow zones with a free surface slope exceeding 10°. The correlation between the digitalized free surface data and the flow depth measured by the instruments was also found to be accurate with errors of ±0.2 mm. The uncertainty of this dataset falls within tolerable ranges for hydro-environmental studies that used the free surface data for model validation. In fact, some of the free surface data from the steady hydraulic jump experiments of this work were used for model validation by Castro-Orgaz *et al*.^22^ using a high-order depth-averaged fluvial model, resulting in excellent agreement. These results by Castro-Orgaz *et al*.^22^ were achieved by assuming a unique Manning’s roughness coefficient *n* = 0.01 for the roughness of both bed surfaces in the flume, namely the stainless steel Gaussian-shape obstacle and the flume floor with the autolevelling two-component polyurethane coating. This suggests the use of this value for validating numerical models using the datasets provided with this manuscript. This indicates the suitability and accuracy of the dataset provided here. Figures 14, 15 shows the results of the digitalized free surface data at different time instants in various experiments in this work. Clear data portions at the 2 beams joints in the flume are apparent in Fig. 15a. Complete plots for the digitalized instants are available in^24^ through the data packages.

### Pressure data

**Fig. 16:**
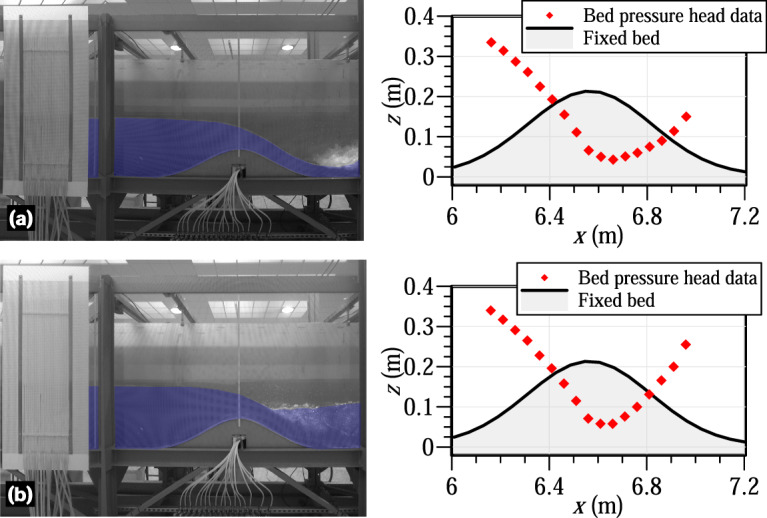
Bed pressure data along the Gaussian-shaped bed profile for the steady conditions: (**a**) experiment 1 and (**b**) experiment 2.

The main source of uncertainty in determining the bed pressure through the piezometers, installed along the bed obstacle, was found to be the accuracy of the readings. This process was conducted manually using a millimetre panel installed in the background of the piezometric panel. Due to the water surface tension effect inside the piezometric tubes producing a meniscus), the piezometric level reading was taken in the axis of the tube. To check the accuracy of the readings, several tests were conducted using still water in the flume. The flow depth was measured using a point gauge (±0.1 mm ruler accuracy) and the readings were then compared with the piezometric readings after deducting the reference level (as explained in the preceding sections). This validation technique resulted in excellent agreement between the two sets of readings with R^2^ over 0.97. Furthermore, we used the projection of the water surface line recorded by the monitoring cameras in various still water and steady flow experiments to verify the correspondence with the piezometric level. It was expected that both levels would match if the piezometric tubes were functioning correctly. Through this validation technique, we obtained satisfactory results, further confirming the reliability of the piezometric tubes for measuring the bed pressure along the Gaussian-shaped bed profile. Figure 16 shows the bed pressure data along the Gaussian-shaped bed profile for the steady hydraulic jump experiments 1 and 2, provided for illustration purposes. Part of this pressure data was used for validation of a high-order depth-averaged fluvial flow model by the first and second authors of this study^22^, yielding excellent simulation results.

## Data Usage

The availability of detailed unsteady and steady data for hydraulic jump experiments in open-channel flow, particularly involving instream obstacles, is scarce in the literature. However, accurate data for the flow variables in these hydraulic phenomena are crucial for the development and validation of physically-based simulation tools in fluvial settings, which are key for risk-assessment studies. Conducting river field studies to accurately measure the variables such as flow depth and bed pressure levels, can be challenging and costly. Thus, we alternatively conducted a laboratory-controlled study with a series of unique experiments designed to investigate steady and unsteady open-channel flows with hydraulic jumps interacting with uneven bathymetry, i.e., an instream obstacle. The database provided in this study will enable scientists to validate fluvial models, enhancing the accuracy of free surface flow descriptions under non-hydrostatic and turbulent conditions—both pivotal areas in contemporary research^1,2,5–7,13,14,21–23^. While aerated flows were observed in the experiments, they are not within the scope of this study.

The experiments provided accurate measurements of the free surface level in a large flume domain and the bed pressure along the bed obstacle^24^. The measured datasets for the free surface flow were obtained using a multi-step process approach involving image acquisition, postprocessing and digitalization. The bed pressure datasets were obtained using classical piezometers. Additionally, the images used in the multi-step process for generating the free surface data are provided, enabling modellers to create unique datasets with the desired degree of detail. Codes and data-processing workflows are also supplied to enhance the understanding and utilization of the data. To the authors’ knowledge, there is not a similar dataset available in the literature. The datasets introduced in this study are valuable for the following purposes:

- **Higher-order shallow water fluvial modelling:** The data of the free surface position under steady and unsteady conditions along with the bed pressure data can be used to validate non-hydrostatic shallow water models of use in fluvial hydraulics. The vertical flow field significantly develops in the vicinity of the bed obstacle, deviating the pressure distribution from the hydrostatic law and affecting the water levels both upstream and downstream. The provided bed pressure data are particularly relevant for the validation of non-hydrostatic models.
- **Turbulence modelling in open-channel flows:** The accurate experimental characterization of steady and unsteady hydraulic jumps provided in this work can be used to develop, calibrate, and validate turbulence models for river flow simulations. Moreover, the provided bed pressure data, including measurements at the beginning of the hydraulic jumps in some tests (those with submerged jumps over the bed obstacle), offers a unique combination of free surface and bed pressure data for hydraulic jumps over uneven beds.
- **Enhancement of laboratory experimental methods and procedures:** The accurate definition of the methods employed in the experimental campaigns presented in this study allows to replicate the laboratory conditions, to expand the provided database, and to improve methods and procedures. Moreover, since the raw images are provided as a part of the outcomes of this study, researchers can study alternative postprocessing methods to enhance the data extraction, such as those aiming at extracting detailed and continuous free surface profiles.

## Data Availability

The codes used for the data-processing in this study are provided in the Zenodo repository publication^24^.
